# Supplementary material for: Information and Communication Technologies in Lung Transplantation: Perception of Patients and Medical Teams
Source: Pharmacy (Basel). 2022 Jun 30;10(4):75. doi: 10.3390/pharmacy10040075 (PMC9326681; doi:10.3390/pharmacy10040075)
Supplement: Supplementary file 1 [file pharmacy-10-00075-s001.zip › pharmacy-1758037-supplementary.pdf]

Table S1. 15 multiple response questions.

Q1	Reminder for healthcare appointments in pneumology department
Q2	Reminder for the shifting of tacrolimus intake to 8pm the eve of the dosage
Q3	Information of a change in dosage of tacrolimus
Q4	Information sending a new prescription (to the patient or fax to the pharmacy)
Q5	Information on the need of a blood sample to do in the laboratory city
Q6	Information sending a new prescription (to the patient or fax to the laboratory)
Q7	Information from the prescription of a new drug
Q8	Reminder for daily immunosuppressive drug intake
Q9	Reminder for daily others drug intake
Q10	Reminder for punctual drug intake
Q11	Reminder for punctual healthcare appointments
Q12	Reminder for get vaccinated against influenza
Q13	Reminder for do and transmit to the measures of breath with Spirotel®
Q14	Information stopping certain medications before an exam
Q15	Means of communication and questions / answers patients / medical team
